# Nuclear medicine technologists are able to accurately determine when a myocardial perfusion rest study is necessary

**DOI:** 10.1186/1472-6947-12-97

**Published:** 2012-09-04

**Authors:** Elin Trägårdh, Liselott Johansson, Camilla Olofsson, Sven Valind, Lars Edenbrandt

**Affiliations:** 1 Department of Clinical Physiology and Nuclear Medicine, Skåne University Hospital, Lund University, Inga Marie Nilssons gata 49, 205 05, Malmö, Sweden

**Keywords:** Image interpretation, Radionuclide imaging, Ischemic heart disease, 99Tc MPS

## Abstract

**Background:**

In myocardial perfusion scintigraphy (MPS), typically a stress and a rest study is performed. If the stress study is considered normal, there is no need for a subsequent rest study. The aim of the study was to determine whether nuclear medicine technologists are able to assess the necessity of a rest study.

**Methods:**

Gated MPS using a 2-day 99mTc protocol for 121 consecutive patients were studied. Visual interpretation by 3 physicians was used as gold standard for determining the need for a rest study based on the stress images. All nuclear medicine technologists performing MPS had to review 82 training cases of stress MPS images with comments regarding the need for rest studies, and thereafter a test consisting of 20 stress MPS images. After passing this test, the nuclear medicine technologists in charge of a stress MPS study assessed whether a rest study was needed or not or if he/she was uncertain and wanted to consult a physician. After that, the physician in charge interpreted the images and decided whether a rest study was required or not.

**Results:**

The nuclear medicine technologists and the physicians in clinical routine agreed in 103 of the 107 cases (96%) for which the technologists felt certain regarding the need for a rest study. In the remaining 14 cases the technologists were uncertain, i.e. wanted to consult a physician. The agreement between the technologists and the physicians in clinical routine was very good, resulting in a kappa value of 0.92. There was no statistically significant difference in the evaluations made by technicians and physicians (P = 0.617).

**Conclusions:**

The nuclear medicine technologists were able to accurately determine whether a rest study was necessary. There was very good agreement between nuclear medicine technologists and physicians in the assessment of the need for a rest study. If the technologists can make this decision, the effectiveness of the nuclear medicine department will improve.

## Background

Stress myocardial perfusion scintigraphy (MPS) is widely regarded as a clinically useful non-invasive imaging modality for diagnosing patients with suspected coronary artery disease [1-3]. Worsley et al [4] demonstrated that rest images were not required if normal imaging findings had been obtained after exercise or pharmacologic stress. The same results have been confirmed by others [5]. Current guidelines also recommend the stress study to be performed first, since the rest study can be omitted if the stress study is interpreted as normal. Thus a rest study should only be performed in patients with equivocal or clearly abnormal studies [6]. The advantages of such an approach are to substantially reduce radiation exposure, lower costs by eliminating unnecessary imaging time and radiopharmaceutical doses, and improve laboratory efficiency by freeing up camera time to study additional patients.

Chang et al [7] investigated whether a normal stress-only MPS confers the same prognosis as a normal MPS on the bases of evaluation of stress and rest images. They found that patients who had a normal MPS on the basis of stress imaging alone have a similar mortality rate as those who have a normal MPS on the basis of evaluation of both stress and rest images.

Nuclear medicine technologists usually review the quality of MPS images, for example signs of patient motion or high extra-cardiac uptake. The assessment of whether a rest study is needed is usually made by a physician. If this could be delegated to the technologist who acquires the stress images, clinic workflow could improve. The aim of the present study was to determine whether nuclear medicine technologists are able to determine the need for a rest study.

## Methods

### Education of nuclear medicine technologists

102 patients admitted to MPS in 2010 were selected for education of nuclear medicine technologists before the start of the study. All stress studies were interpreted by two experienced physicians who judged each study as “no rest study necessary” or “rest study necessary”. When there was disagreement between the physicians, a third physician evaluated the studies. Thus, agreement between 2 out of 3 physicians was considered gold standard. Attenuation-corrected (AC), non-attenuation corrected (NC) stress images and gated images were available for this interpretation. 82 of the cases were selected as training cases. The nuclear medicine technologists were exposed to stress-only images including information about the gated studies, then stress and rest images and then a comment about the study (why a rest study was necessary or not in a particular case). The nuclear medicine technologists were educated in the same manner as new physicians in our department when evaluating the need for a rest study. This includes that in order to be considered as “completely normal” or “probably normal” study (i.e. no rest study required), the perfusion intensity had to be so high in the stress image of either the NC or the AC images, that no matter the appearance of the rest study, the study would still not be considered as having a defect. The ejection fraction also had to be normal. 20 studies were then selected as test cases. The nuclear medicine technologists evaluated stress-only images in these patients, and categorized the images as “rest study required” or “no rest study required”. One mistake or less was considered as a passed test. All nuclear medicine technologists passed the test.

### Study population

The study group consisted of 130 patients admitted to MPS between April and June 2011 at Skåne University Hospital, Malmö, Sweden. Of these, 9 patients were excluded due to missing MPS files or missing data in the evaluation forms. Mean age was 54 ± 8 years; 49% were men. The study was performed in accordance with the principles of the Declaration of Helsinki. The Ethics Committee at Lund University made an advisory statement in which it considered that there was no objection against it from an ethics point of view.

### Radionuclide imaging

The MPS studies were performed using a 2-day gated stress/non-gated rest Tc-99m-tetrofosmin protocol, starting with injection of 600 MBq Tc-99m-tetrofosmin at stress. Patients were stressed using either maximal exercise on an ergometer or pharmacological test with adenosine. The exercise was continued for at least 1 min after the injection of the tracer and the adenosine infusion at least 2 min after the injection of the tracer. Normal findings at stress were not followed by a rest study. Not definitely normal stress studies were followed by a rest study with injection of 600 MBq Tc-99m-tetrofosmin.

Stress and rest acquisition began about 60 min after the end of the injection of Tc-99m-tetrofosmin. Images were obtained according to established clinical protocols, using SPECT over 180 ° elliptical, autocontour rotations from the 45° right anterior oblique position, with a dual-head gamma camera, e.cam (Siemens AG Medical Solutions, Erlangen, Germany). Patients were imaged in the supine position. Low energy high-resolution collimator and a zoom factor of 1.0 were used. We obtained 64 (32 views per camera) projections in a 128 x 128 matrix, with an acquisition time of 25 s per projection. Stress images were gated to the electrocardiogram using 8 frames per cardiac cycle. No automatic motion-correction program was applied; instead the acquisition was repeated if motion was detected. Tomographic reconstruction and calculation of short and long axis slice images were performed using e.soft (Siemens AG Medical Solutions, Erlangen, Germany). Non-attenuation corrected images were reconstructed with filtered back-projection. A 2D Butterworth pre-reconstruction filter was used with critical frequency of 0.45, order 5. Attenuation corrected images were reconstructed with an iterative algorithm, 6 iterations [8] where a ramp filter was applied on the error projection prior to backprojection. A Butterworth filter with a critical frequency of 0.40, order 5, was applied for regularization. Attenuation maps were generated from simultaneous transmission measurement using a Gd-153 multiple-line source (Siemens AG Medical Solutions, Erlangen, Germany) [9]. The cut-off frequencies of the filters were selected so that the noise level in the AC images was similar to that in the NC images.

### Gold standard

All stress studies were interpreted by two physicians (one resident in nuclear medicine with 2 years of clinical and research experience with MPS, and one professor with more than 20 years of clinical and research experience with MPS) who judged each study as “no rest study necessary” (completely normal or probably normal studies) or “rest study necessary” (equivocal, probably or certain abnormal studies). When there was disagreement between the physicians (which happened in 10 cases), a third physician (a senior consultant with more than 20 years of clinical experience with MPS) evaluated the studies, as described above (Education of nuclear medicine technologists). AC, NC images and gated images were available for this interpretation.

### Stress-only assessment

For each study, the technologist responsible for the patient assessed whether a rest study was necessary or not, before consulting the physician in charge. They could choose between the alternatives “rest study required”, “rest study not required” or “uncertain”, i.e. consult a physician. After the technologist made her/his decision, a physician evaluated the images and decided whether a rest study was needed or not. In total, 12 nuclear medicine technologists and 7 physicians were involved in the study. The nuclear medicine technologists had at least 6 months of experience from MPS. Four of the physicians were specialists in nuclear medicine, and 3 were residents in nuclear medicine, with at least 1.5 years of experience from MPS. The technologists and physicians evaluated the images using the e.soft (Siemens AG Medical Solutions, Erlangen, Germany) software. If desired, EXINI heart^TM^ software package (EXINI Diagnostics AB, Lund, Sweden) could also be used for evaluation, both for physicians and technologists. This computer-assisted diagnosis software presents advice regarding normality of a stress study. All observers had access to clinical information.

### Inter- and intra-observer variability

To give an idea about inter- and intra-observer variability for physicians, the gold standard was used. For intra-observer variability, the assessments for the two physicians who created the gold standard were used. One of the physicians (the resident) evaluated all images twice. The evaluations were blinded to one another and were performed approximately 4 months apart. This was used for assessment of inter-observer variability.

Inter-and intra-observer variability for nuclear medicine technologists was also investigated. Two technologists evaluated all stress images, and one of the technologists evaluated all stress images twice. The evaluations were blinded to one another and were performed 1 week apart. The technologists had 4 and 5 years of experience of MPS, respectively. For this assessment, the technologists only had the options “rest study necessary” and “no rest study needed” (not “consult a physician”).

### Follow-up

The new routine was implemented in mid-November 2011. January-October 2011 (10 months) were used as reference (physicians made decisions about the need for a rest study or not). December 2011- March 2012 (4 months were decisions were made by nuclear medicine technologists) were compared to the reference period. The number of stress and rest studies for each period was calculated.

### Statistical methods

The McNemar test was used to analyze the difference in classification of patients into the rest-study-required and no-rest-study-required groups between the nuclear medicine technologists and the physicians. Kappa statistics were used to evaluate the agreement between technologists and physicians, as well as for inter- and intra-observer variability. Fisher’s exact test was used to evaluate difference in the number of rest studies needed before and after the new routine was implemented. P values of less than 0.05 were considered statistically significant. Statistics were carried out using Analyse-it® for Microsoft Excel (Analyse-it Software Ltd, Leeds, UK).

## Results

### Comparison between physicians and nuclear medicine technologists

**Figure 1:**
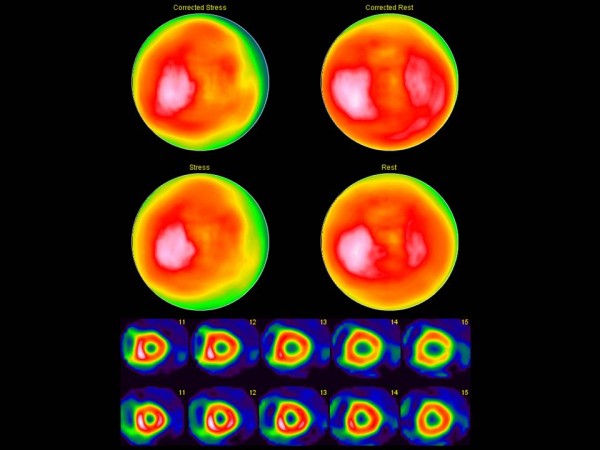
**The figure shows AC stress (left) and rest (right) polar plots (upper row), NC stress and rest polar plots (middle row) and mid-ventricular to basal short axes images (stress above rest; lower row) for the study evaluated as “no rest study needed” by a nuclear medicine technologist.** The study was interpreted as ischemic in the final report.

The nuclear medicine technologists and the physicians in clinical routine agreed in 103 of the 107 cases (96%) for which the technologists felt certain regarding the need for a rest study. There was no statistically significant difference in the evaluations made by technologists or physicians (P = 0.617). In the remaining 14 cases the technologists were uncertain, i.e. wanted to consult a physician. There was disagreement in only 4 cases; 2 of which the technologists wanted a rest study and the physicians did not, and 2 vice versa. For one of the studies where the technologist did not want a rest study, but the physician did, the diagnosis of ischemia was stated in the final report according to clinical routine. Figure 1 shows images from this patient. In the other case, no ischemia or infarction was stated in the final report. In 43 cases both technologists and physicians wanted a rest study, and in 60 cases they agreed that no rest study was necessary. The agreement between the technologists and the physicians in clinical routine was very good resulting in a kappa value of 0.92 for the 107 cases.

The three physicians (gold standard) determined that 73 patients did not require a rest study and 48 patients did require a rest study. For the 107 patients (excluding uncertain cases), the nuclear medicine technologists wished for a rest study in 7 of the cases where it was not necessary according to gold standard, and did not wish a rest study in 5 cases, where a rest study should have been performed. For the physicians in clinical routine, 8 patients performed a rest study when it was not necessary according to gold standard, and in 5 cases a rest study was not performed, where a rest study should have been done. Table 1 shows the distributions for the nuclear medicine technologists and physicians. The overall accuracy was 88.8% for the nuclear medicine technologists and 89.3% for the physicians. Sensitivity, specificity, negative and positive predictive values are presented in Table 2.

**Table 1:** The distribution of the evaluations from the nuclear medicine technologists (A) and physicians (B)

		**Gold standard**		
		**Rest**	**No rest**	
A				
Technologists	Rest	38	7	**45**
	No rest	5	57	**62**
		**43**	**64**	**107**
B				
Physicians	Rest	43	8	**51**
	No rest	5	65	**70**
		**48**	**73**	**121**

Missing data: 14 cases (classified as “uncertain” by technologists).

**Table 2:** Sensitivity, specificity, positive and negative predictive values for the need of a rest study for technologists and physicians

	**Technologists**	**Physicians**
Sensitivity	88%	90%
Specificity	89%	89%
PPV	84%	84%
NPV	92%	93%

PPV – positive predictive value; NPV – negative predictive value.

### Inter- and intra-observer variability

The two physicians who created the gold standard agreed in all cases but 10 (the resident wanted a rest study in 4 cases where the professor did not), resulting in a kappa value of 0.83. The third physician, who evaluated these 10 cases blinded to the results from the other physicians, agreed with the resident in 5 of the cases and with the professor in 5 of the cases. The resident who evaluated all stress images twice, agreed in all but 11 of the cases (wanting a rest study in 5 cases and no rest study in 6 cases when evaluating the second time as opposed to the first time), resulting in a kappa value of 0.81. Six of the 11 cases where the evaluations made by the resident differed between evaluations were the same cases where the two physicians differed, indicating borderline cases.

The nuclear medicine technologists agreed in all cases but 18, resulting in a kappa value of 0.70. The technologist who evaluated all stress images twice, agreed in all but 13 of the cases, resulting in a kappa value of 0.78. There were no cases with ischemia or infarction according to the final report, where the technologists did not requested a rest study.

### Follow-up

For the reference period, in total 1141 patients were examined by MPS, of which 641 also performed a rest study (56.2%). For the period when decisions were made by nuclear medicine technologists, 553 MPS studies were performed, of which 312 had a rest study included (56.4%) (P = 0.96).

## Discussion

We found that nuclear medicine technologists were able to determine when a rest study was required. There was very good agreement between the technologists and the physicians, and the proportion of rest studies did not change after the new routine was introduced.

If the nuclear medicine technologist who acquires the stress image is able to determine the necessity for a rest study, this will improve clinical workflow. The technologist does not have to wait for a decision made by the physician, and the physician will have more time to correctly interpret the images and write the final report to the referring clinician. The present study indicates that it is possible to delegate this assessment to the nuclear medicine technologist. It is, however, important to state that the physicians are still responsible for the final evaluation of the studies. There is always the opportunity to call the patient back for a rest study, if the physician who interprets the images and writes the final reports so desires. All patients that were sent home after the stress study during the follow-up period were informed that a rest study might still be needed and that they would be contacted if a rest study was desired by the physician. Thus, in our opinion this approach increases laboratory efficiency without increasing the risk for false negative interpretations.

In this study, one patient would have been sent home by the nuclear medicine technologist without a rest study, when the study was interpreted as ischemic on the final report. It is not clear whether this was a typing error made by this experienced technologist or if the technologist was not properly trained. If this would have been a patient sent home by a technologist, the patient would have been contacted for a subsequent rest study when the physician responsible for interpreting the study evaluated the images. This patient was not missed by the two technologists who assessed inter- and intra-observer variability.

Inter- and intra-observer variability was higher for technologists than for physicians. This is probably due to less experience of interpreting images for nuclear medicine technologists, and will probably improve over time. In this study, the option of “consulting a physician” was not possible for the inter- and intra-observer variability assessment, which probably also lowered the kappa value.

A similar study was performed by Johansson et al [10] in 2008. In their study, visual interpretation of 532 patients of the complete stress and rest images by 1 experienced physician was used as gold standard. All cases categorized as infarction or ischemia present were categorized as the group requiring a rest study, whereas all other cases were categorized as the group not requiring a rest study. A total of 3 nuclear medicine technologists and 3 physicians independently classified each of the stress studies as rest-study-required or no-rest-study-required. They found that the nuclear medicine technologists were able to assess whether a rest study was needed. The risk that this assessment would be incorrect was not higher for the technologists than it was for the physicians. Their gold standard differed from the one used in the present study. In the clinical routine, more patients than those with ischemia or infarction onthe final interpretation will undergo a rest study (i.e. patients with equivocal images). We therefore believe that our approach is more suitable as a gold standard. Our study with only the technologist and physician responsible for the patient evaluating the need for a rest study also reflects a more “accurate” clinical situation compared to using only 3 technologists and 3 physicians evaluating all images evaluating images only in a retrospective study.

There are both advantages and disadvantages for using a stress-only approach for stress studies interpreted as normal or probably normal. In most cases, the approach will improve work flow in the nuclear medicine department and, more importantly, reduce the radiation dose to the patient. However, there is a possibility that small defects might be overlooked at the stress study, thus giving false negative interpretations. There is also a possibility that balanced 3-vessel disease only presenting with transient ischemic dilatation and reduction in ejection fraction on stress images are missed when no rest studies are available for comparison. Current guidelines, however, recommend that rest studies should not be performed if the stress study is considered normal [6].

### Study limitations

The physicians who evaluated the NC and AC stress studies for the gold standard did not have any clinical information about the patients. It is possible that there would have been higher agreement between gold standard and technologists/physicians if the clinical information would have been available when deciding the gold standard.

## Conclusions

There was very good agreement between nuclear medicine technologists and physicians in the assessment of the need for a rest study. The proportion of rest studies did not change in the follow-up period after the new routine was introduced. If the technologists can make this decision, the effectiveness of the nuclear medicine department will improve.

## Abbreviations

AC, Attenuation-correction; MPS, Myocardial perfusion scintigraphy; NC, Non-attenuation correction.

## Competing interests

LE is a stockholder of EXINI Diagnostics AB.

## Authors’ contributions

ET participated in the design of the study, performed the statistical analysis and drafted the manuscript. LJ and CO participated in the design of the study and coordination. SV and LE participated in the design of the study and helped to draft the manuscript. All authors read and approved the final manuscript.

## Financial support

This work was supported by grants from the Faculty of Medicine, Lund University, Sweden.

## Pre-publication history

The pre-publication history for this paper can be accessed here:

http://www.biomedcentral.com/1472-6947/12/97/prepub
